# Enhancing Workforce Readiness for Aging and Disability Networks: A No Wrong Door State Training Initiative

**DOI:** 10.1093/geroni/igaf122.1633

**Published:** 2025-12-31

**Authors:** Bronwyn Keefe, Annalee Wilson, Erin Verdile

**Affiliations:** Boston University School of Social Work, Boston, Massachusetts, United States; Boston University, Boston, Massachusetts, United States; New York State Office for the Aging, Albany, New York, United States

## Abstract

This project focused on enhancing the competencies of information and assistance specialists and options counselors working in New York’s No Wrong Door (NWD) system by providing innovative, skill-based online training. Responding to a need for specialized person-centered training, the New York State Office for the Aging (NYSOFA) and the Center for Aging and Disability Education and Research (CADER) at Boston University partnered with the goals of developing and implementing a standard training program for NWD staff, and ensuring that staff have the knowledge and skills to support older adults. 465 learners completed the training, which included coursework in aging and disability issues and networks, performing assessments, person-centered planning, mental health, and ethics. Two customized courses were developed: a course describing resources for older adults offered by three NY state offices, and a video scenario course where actors depicted situations that NWD staff may encounter. Learners self-reported their skill level on course competencies before and after each course and showed statistically significant (p < 0.05) improvements on all competencies. On a pre-post workforce readiness assessment, which included statements such as “I know how to review eligibility criteria for various resources,” there was a statistically significant (p < 0.05) change in learners’ agreement with 18 out of the 20 statements, indicating that the training had a positive impact on their ability to effectively support older adults navigating the NWD system. Future directions include the development of a state policy requiring that NWD staff complete the training program upon hire and recertify every three years.

